# Radiologist observations of computed tomography (CT) images predict treatment outcome in TB Portals, a real-world database of tuberculosis (TB) cases

**DOI:** 10.1371/journal.pone.0247906

**Published:** 2021-03-17

**Authors:** Gabriel Rosenfeld, Andrei Gabrielian, Qinlu Wang, Jingwen Gu, Darrell E. Hurt, Alyssa Long, Alex Rosenthal

**Affiliations:** 1 Bioinformatics and Computational Biosciences Branch, Office of Cyber Infrastructure and Computational Biology, National Institute of Allergy and Infectious Diseases, National Institutes of Health, Bethesda, MA, United States of America; 2 Software Engineering Branch, Office of Cyber Infrastructure and Computational Biology, National Institute of Allergy and Infectious Diseases, National Institutes of Health, Bethesda, MA, United States of America; 3 Office of Cyber Infrastructure and Computational Biology, National Institute of Allergy and Infectious Diseases, National Institutes of Health, Bethesda, MA, United States of America; Jamia Hamdard, INDIA

## Abstract

The TB Portals program provides a publicly accessible repository of TB case data containing multi-modal information such as case clinical characteristics, pathogen genomics, and radiomics. The real-world resource contains over 3400 TB cases, primarily drug resistant cases, and CT images with radiologist annotations are available for many of these cases. The breadth of data collected offers a patient-centric view into the etiology of the disease including the temporal context of the available imaging information. Here, we analyze a cohort of new TB cases with available radiologist observations of CTs taken around the time of initial registration of the case into the database and with available follow up to treatment outcome of cured or died. Follow up ranged from 5 weeks to a little over 2 years consistent with the longest treatment regimens for drug resistant TB and cases were registered within the years 2008 to 2019. The radiologist observations were incorporated into machine learning pipelines to test various class balancing strategies on the performance of predictive models. The modeling results support that the radiologist observations are predictive of treatment outcome. Moreover, inferential statistical analysis identifies markers of TB disease spread as having an association with poor treatment outcome including presence of radiologist observations in both lungs, swollen lymph nodes, multiple cavities, and large cavities. While the initial results are promising, further data collection is needed to incorporate methods to mitigate potential confounding such as including additional model covariates or matching cohorts on covariates of interest (e.g. demographics, BMI, comorbidity, TB subtype, etc.). Nonetheless, the preliminary results highlight the utility of the resource for hypothesis generation and exploration of potential biomarkers of TB disease severity and support these additional data collection efforts.

## Introduction

TB is a global pandemic resulting in approximately 9 million new cases and 1.5 million deaths each year [1]. The emergence of drug resistance where up to ~20% of TB isolates globally are estimated to be resistant to a major drug [2] threatens to exacerbate the pandemic especially the emergence of totally drug-resistant TB now endemic in specific countries. Out of cases that are not totally drug resistant, drug resistance varies from mono resistant to a first line drug to extensively drug resistant (XDR) to isoniazid and rifampin, as well as any fluoroquinolone and one or more of three injectable second-line drugs (i.e., amikacin, kanamycin, or capreomycin). Drug resistance is associated with poorer outcomes and higher costs of care compared to drug sensitive TB with treatment success at ~55% globally and Multi- or Extensively Drug-resistant TB (M/XDR-TB) having a cost of care up to 25 times that of drug sensitive cases [3,4].

CT imaging is routinely collected during the management of TB to assess patient disease status [5]. Moreover, the use of mobile radiology can improve detection and screening of TB cases in harder to reach populations [6]. Such distributed approaches support distant diagnosis and remote monitoring of disease severity through the analysis of the resulting data via machine learning and other emerging approaches. Radiologist observations are the gold standard reference upon which CT images have been interpreted for clinical insights and actionable information historically [7]. These observations contain pertinent insights to inform patient risk and may have less of a barrier to interpretation than emerging approaches such as deep learning since they are often captured in a common, clinical vernacular. Prior research has demonstrated the utility of radiologist observations from images for assessing patient risk. For example, CT scans were predictive of treatment outcome [8,9], bilateral lung involvement in active TB showed higher risk of underlying diabetes millitis [10], and pulmonary TB patients with chest CT findings of cavity, consolidation, bronchiectasis, upper lobe involvement, multiple lobe involvement, and lymphadenopathy indicated a higher risk for smear-positive TB [11].

The Office of Cyber Infrastructure and Computational Biology established the TB Portals program as an international collaboration to support TB data sharing and data science facilitating the biomedical research community’s efforts to understand the real-world impact of TB [12]. The TB Portals program contains a publicly available repository of TB case data capturing multi-modal information such as case clinical characteristics, pathogen genomics, and radiomics that can provide a unique understanding of TB disease etiology over time. As of November 2020, the TB Portals resource contains over 3400 TB cases, primarily drug resistant cases, many of which contain associated CT images with radiologist annotations. While other clinical image resources exist with large numbers of images, TB Portals offers a patient-centric resource that captures the temporal context of each case associated with the CT images including drug resistance status of the case, the drugs administered, and the pathogen identified. External collaborators can request data access through a data use agreement (DUA) and download publicly shared data supporting reproducibility and open-science.

In this study, we sought to leverage the available radiologist observations for CT images in the TB Portals repository to assess their utility for predicting patient treatment outcome independent of other case characteristics or data modalities the resource provides. We examined the available radiologist observations from CTs close to the initial registration of the case into the database and identified the most important variables that are predictive of treatment outcome. We used the quarterly updated published data available to external collaborators from October 2020 to create a cohort of new cases of TB having the following inclusion and exclusion criteria: an initial annotated CT record within 60 days of the first sample recorded in the database, a treatment outcome of “cured” or “died”, and follow up from CT record to treatment outcome greater than 0 weeks. This cohort was used for a retrospective, case-control study assessing presence of various radiologist observations towards risk of treatment outcome of died. As we observed ~10% of treatment outcomes resulting in “died”, we compared class balancing approaches to increase the representation of these clinically relevant cases and assessed impact on the performance of the predictive models to detect this outcome. We also generated inferential statistics on the risk of outcome of death associated with these radiologist observations. Since the TB Portals constitutes real-world data, it can be difficult to decouple the risks with other underlying characteristics of the cases. Nonetheless, we believe that the findings from this study identify radiological signals that may indicate a problematic case or biomarkers that could inform clinical trial design as markers of disease severity. Moreover, these observations confirm prior findings showing the association of cavitary disease with poor treatment outcomes.

## Materials and methods

### Computing environment

All analyses were done on a MacBook Pro laptop (x86_64-apple-darwin15.6.0 (64-bit) Running under: macOS Mojave 10.14.6) using R version 4.0.2 (2020-06-22) and RStudio 1.2.5033. Specific R packages versions used can be found in S1 Fig.

### Cohort selection

New cases of TB with available CT images and treatment outcome of “cured” or “died” were identified in R using publicly available data from quarter 3 of 2020 that is available to external collaborators after signing a DUA (see Data Availability methods section). The external data files were downloaded via aspera service and loaded in R. The exclusion/inclusion criteria were applied using coding logic that can be found in the following GitHub repo (https://github.com/niaid/tbportals.ct.analysis.2020) as a drake workflow for reproducibility. We identified a cohort of 371 new cases of TB with an available CT annotation and a treatment outcome of “cured” or “died”. Application of subsequent inclusion/exclusion criteria including first available CT with radiologist annotation, follow up to the ending of the treatment period of greater than 0 weeks, and CT date within 60 days of registration reduced the number of cases to 253. 228 cases had an outcome of cured and 25 cases had an outcome of died. Case characteristics were compared by treatment outcome in S1 Table.

### Data preprocessing for benchmarking model performance

The cohort contained radiologist observations with either no variance between cured or died groups or only one or zero cases in a particular factor level within a comparison group as shown in S2 Table; therefore, we removed any annotations with limited variation or recoded covariates incorporating the feedback from a TB disease expert in order to increase statistical power within the subgroups. Specifically, bodysite_coding_cd variable combined Left lung and Right Lung categories into One Lung category; lungcavitysize variable combined 10-25mm and Less than 10mm categories into the LTE 25mm; affectlevel variable combined Lower Lobus, Medium and Lower Lobbi, Upper and Lower Lobbi, and Upper and Medium Lobbi into Lower or medium category; and totalcavernum combined 1 cavity and 2 cavities variables into LTE to 2 cavities variable. The radiologist observations after initial preprocessing demonstrated statistically significant differences in observations between cases according to treatment outcome as shown in S3 Table. Moreover, correlations between covariates suggested associations that reflected clinical observation of disease severity and indicated potential predictive capability as seen in S2 Fig. Dropping or refactoring of variables were completed before running the rest of the data preprocessing steps, which were incorporated into an Mlr3 [13] pipeline for an unbiased assessment of the subsequent preprocessing steps on performance via 5-fold cross-validation. The subsequent preprocessing steps included top 5 features selection via mutual information, encoding of features to binary indicator format, removal of any zero variance encoded features within a cross-validation split, random sampling to replace any missing data, standardization of factors that were missing levels in a particular split. Class balancing involved the use of Mlr3’s default class balancing where the majority and minority class were brought to an even proportion through a combination of upsampling and downsampling or the SMOTE algorithm for synthetic generation of minority class examples. MLR3 implementation of the SMOTE algorithm uses numerical data and can lead to synthetic data having intermediary values between 0 and 1 for the set of binary features used. Synthetic data created by SMOTE was rounded to 0 or 1 to avoid data leakage where the model can learn to identify synthetic data and its connection to the outcome of died.

### Benchmarking model performance

Mlr3 R package was used to generate a pipeline of preprocessing steps and downstream machine learning algorithms for performance benchmarking. Data was split 75% and 25% into a training and validation set respectively for benchmarking and validation of prediction performance respectively. For binary classification, pipelines were benchmarked with or without class balancing to increase the representation of the rarer class of “died” constituting only ~10% of cases. For binary classification, model performance was compared to a featureless model that predicted the class with the most observations in the training split or a random selection in case of a tie. The selection of binary classifier models assessed included a featureless model (https://mlr3.mlr-org.com/reference/mlr_learners_classif.featureless.html), logistic regression (https://mlr3learners.mlr-org.com/reference/mlr_learners_classif.log_reg.html), weighted k-nearest neighbors (https://mlr3learners.mlr-org.com/reference/mlr_learners_classif.kknn.html), multinomial log-linear learner via neural networks (https://mlr3learners.mlr-org.com/reference/mlr_learners_classif.multinom.html), and random forest (https://mlr3learners.mlr-org.com/reference/mlr_learners_classif.ranger.html). For time-to-event benchmarking, the time variable of weeks from CT to treatment outcome was included to model the time to death of the right-censored data. Censoring of cured patients occurred at the treatment period end date. Three survival models were tested including Kaplan-Meier estimator (https://mlr3proba.mlr-org.com/reference/mlr_learners_surv.kaplan.html), cox proportional hazards (https://mlr3proba.mlr-org.com/reference/mlr_learners_surv.coxph.html), and decision tree (https://mlr3proba.mlr-org.com/reference/mlr_learners_surv.rpart.html). Harrell’s C-statistic was used to assess survival model performance and multiple measures were used to assess binary classifier performance.

### Calculation of inferential statistics

Odds ratios and hazard ratios were calculated using all available data and the R package finalfit. To select the top 5 most important features for multivariate modeling, mutual information feature selection was applied on the entire dataset. All covariates were tested using univariate modeling while only the top 5 features were included in the multivariate models. Multiple imputation using 5 independent imputations and standard parameters in the mice R package was performed to generate the multiply imputed multivariate estimates. The proportional hazards assumption was tested using the cox.zph function and confirmed as shown in S4 Table. Top 5 features by mutual information showing any collinearity via variance inflation factor were dropped from the final multivariate model (e.g. total number of cavities and cavity size which share a “No cavities” level that is perfectly correlated).

### Kaplan-Meier curves

Kaplan-Meier curves for covariates were generated using the survminer R package. All plots included a table of observations at each time point to reflect censoring and number of available cases at each time point. Survival probability is plotted along with the 95% confidence intervals.

### Data availability and code

The TB portals requires all users of the data to abide by a DUA before access to the underlying clinical data is provided and the data can be requested at the following URL (https://tbportals.niaid.nih.gov/download-data). Therefore, this study provides the code to reproduce the analysis without the underlying raw data (https://github.com/niaid/tbportals.ct.analysis.2020) in compliance with the DUA. To rerun the analysis, interested parties can request data access by completing the DUA and then place the downloaded clinical data files to the subdirectory of the data folder as provided in the GitHub repo instructions. To add reproducibility, the list of patient and condition identifiers are provided in S5 Table so that those interested in assessing the specific cohort are able to do so after completion of required DUA irrespective of future growth in the database.

## Results

### Inferential statistics associated with poor outcome

The main objective of the study was to understand whether radiologist observations of CT images within TB Portals, independent of other data connected with the case, contained any features associated with risk of poor treatment outcome. The results from our analysis included statistically significant risk factors that are identified with poor outcome. We modeled the radiologist observations by both univariate and multivariate logistic regression focusing on the top 5 important features by mutual information with the treatment outcome. Cavity size and number of cavities were selected by mutual information which was interesting as cavities were associated with established disease and disease severity [14,15] and cavitary disease has been reported previously to be associated with poor treatment outcome in clinical trials [8,9]. Nonetheless, both cavitary features showed a significant level of collinearity that can adversely affect modeling. We dropped total number of cavities from the multivariate model to prevent the observed collinearity affecting estimates. Some radiologist observations contained missing values so we generated multiply imputed data for multivariate modeling to assess impact on estimates. Odds ratio estimates for univariate, multivariate, and multiply imputed multivariate logistic regression were shown (Table 1). In general, cases with observations indicating TB disease spread showed higher odds to develop treatment outcome of died compared to cases without these observations. These biomarkers of disease spread included whether observations were present in both lungs (bodysite_coding_cd), presence of swollen lymph nodes (limfoadenopatia), and whether large cavities were observed greater than 25mm in size (lungcavitysize).

**Table 1:** Odds ratios from univariate, multivariate, and multiply imputed multivariate logistic regression.

Dependent: event	Level	Cured	Died	OR (univariable)	OR (multivariable)	OR (multiply imputed)
**affectlevel**	Upper Lobus	107 (95.5)	5 (4.5)	-	-	-
	Lower or medium	57 (91.9)	5 (8.1)	1.88 (0.50–7.01, p = 0.335)	0.76 (0.17–3.37, p = 0.717)	1.13 (0.27–4.75, p = 0.864)
	Total lung affected	29 (87.9)	4 (12.1)	2.95 (0.69–11.86, p = 0.123)	0.42 (0.07–2.19, p = 0.309)	0.97 (0.21–4.45, p = 0.971)
**affectpleura**	No	63 (90.0)	7 (10.0)	-	-	-
	Yes	164 (90.1)	18 (9.9)	0.99 (0.41–2.64, p = 0.979)	-	-
**bodysite_coding_cd**	one_lung	137 (97.9)	3 (2.1)	-	-	-
	both_lungs	91 (80.5)	22 (19.5)	11.04 (3.69–47.60, p<0.001)	13.18 (2.98–93.65, p = 0.002)	8.86 (2.19–35.78, p = 0.002)
**bronchialobstruction**	No	181 (91.9)	16 (8.1)	-	-	-
	Yes	43 (82.7)	9 (17.3)	2.37 (0.95–5.63, p = 0.055)	-	-
**dissemination**	No	156 (91.8)	14 (8.2)	-	-	-
	Yes	71 (86.6)	11 (13.4)	1.73 (0.73–3.98, p = 0.202)	-	-
**limfoadenopatia**	No	202 (93.5)	14 (6.5)	-	-	-
	Yes	25 (69.4)	11 (30.6)	6.35 (2.57–15.54, p<0.001)	5.67 (1.28–26.19, p = 0.021)	6.32 (1.95–20.46, p = 0.002)
**lungcapacitydecrease**	No	182 (92.4)	15 (7.6)	-	-	-
	Yes	45 (81.8)	10 (18.2)	2.70 (1.11–6.35, p = 0.024)	-	-
**lungcavitysize**	No cavities	142 (92.8)	11 (7.2)	-	-	-
	LTE to 25mm	77 (89.5)	9 (10.5)	1.51 (0.58–3.80, p = 0.383)	2.22 (0.56–9.41, p = 0.257)	2.59 (0.81–8.27, p = 0.106)
	More than 25mm	8 (61.5)	5 (38.5)	8.07 (2.14–28.77, p = 0.001)	3.45 (0.14–39.92, p = 0.352)	6.68 (1.40–31.93, p = 0.017)
**nodicalcinatum**	No	207 (90.8)	21 (9.2)	-	-	-
	Yes	20 (83.3)	4 (16.7)	1.97 (0.54–5.82, p = 0.253)	-	-
**plevritis**	No	209 (91.7)	19 (8.3)	-	-	-
	Yes	18 (75.0)	6 (25.0)	3.67 (1.21–9.97, p = 0.014)	-	-
**pneumothorax**	No	224 (91.1)	22 (8.9)	-	-	-
	Yes	2 (40.0)	3 (60.0)	15.27 (2.41–120.75, p = 0.004)	-	-
**posttbresiduals**	No	201 (89.7)	23 (10.3)	-	-	-
	Yes	25 (92.6)	2 (7.4)	0.70 (0.11–2.56, p = 0.641)	-	-
**processprevalence**	Less than 2 segments	102 (96.2)	4 (3.8)	-	-	-
	2 or more segments	125 (85.6)	21 (14.4)	4.28 (1.57–15.04, p = 0.010)	-	-
**totalcavernum**	No cavities	142 (92.8)	11 (7.2)	-	-	-
	More than 2 cavities	21 (67.7)	10 (32.3)	6.15 (2.31–16.41, p<0.001)	-	-
	LTE to 2 cavities	64 (94.1)	4 (5.9)	0.81 (0.22–2.46, p = 0.722)	-	-

All radiologist annotations were used for univariate modeling whereas only the top 5 features selected by mutual information based upon treatment outcome were used for multivariate estimates. For multiple imputation, the MICE algorithm was used with default settings to generate 5 imputed datasets used for calculating estimates that were then pooled for the multiply imputed estimates. Cured and died columns show the number of complete cases having the particular radiologist annotation with percentage of cases with that particular observation having the particular outcome in parenthesis that is available for univariate modeling. While cured or died numbers show complete cases for the univariate estimates, multiply imputed estimates use imputed data for the entire set of data (n = 204 cases). OR columns show the odds ratios for univariate, multivariate, and multiple imputed multivariate estimates respectively with p-values and 95% confidence intervals in parenthesis with upper and lower bounds shown with a dash between. For univariate or multivariate reference levels within a covariate, a dash is used. For variables not used in the multivariate model, a dash is provided. Markers of disease progression such as presence of TB-related annotations in both lungs, swollen lymph nodes, and cavity size show statistically significant higher odds ratios for an outcome of died. Glossary: Affectlevel–location of affected lung area; affectpleura—changes in the pleura; bodysite_coding_cd–which lung is the observation located; bronchialobstruction—bronchial obstruction syndrome disorders, dissemination—Diffuse pulmonary nodules detected; limfoadenopatia–greater than 10 mm is considered the upper limit for normal nodes (short transverse diameter); lungcapacitydecrease—reduced lung volumes; lungcavitysize–size of lung cavity; nodalcalcinatum—Nodi Calcinatum detected; plevritis—pleural effusion detected; pneumothorax—Pneumothorax detected; posttbresiduals—Post-TB changes in the lung; processprevalence–prevalence of process in number of segments; totalcavernum–number of cavities; thromboembolismpulmonaryartery—Thromboembolism Of The Pulmonary Artery detected; anomalymediastinumvesselsdevelop—Anomaly Of Mediastinum Vessels Develop detected; shadowpattern–shadowpattern of nodule, node, or infiltrate; affectedsegments–segments of lung that are affected; accumulationcontrast–amount of contrast accumulated.

To incorporate the temporal aspects of each CT with treatment outcome, we also generated hazard ratio estimates using cox regression and noted similar findings to risks identified in logistic regression models (Table 2). Observations associated with disease spread and activity showed higher hazard ratios for a treatment outcome of died. These included whether observations were present in both lungs and whether large cavities were observed greater than 25mm in size. We noted statistically significant univariate or multiple imputation multivariate risks for lungcavitysize; however, the multivariate model risk did not show statistical significance. The multivariate model uses complete case information for the set of records and variables. Due to combinations of missingness across variables, this decreases the available numbers of complete cases leading to the observed differences in statistical significance for the lungcavitysize variable. Multiple imputation suggests that lungcavitysize would show significant differences controlling for the other features included in the multivariate model but only additional data collection will be able to confirm this in complete cases.

**Table 2:** Hazard ratios from univariate, multivariate, and multiply imputed multivariate cox proportional hazards regression.

Dependent: Surv(time, event)	Level	HR (univariable)	HR (multivariable)	HR (multiply imputed)
**affectlevel**	Upper Lobus	-	-	-
	Lower or medium	1.86 (0.54–6.43, p = 0.326)	1.00 (0.23–4.35, p = 0.996)	0.92 (0.13–6.65, p = 0.918)
	Total lung affected	2.96 (0.79–11.03, p = 0.106)	0.84 (0.16–4.50, p = 0.841)	1.09 (0.26–4.55, p = 0.894)
**affectpleura**	No	-	-	-
	Yes	0.96 (0.40–2.30, p = 0.931)	-	-
**bodysite_coding_cd**	one_lung	-	-	-
	both_lungs	10.04 (3.00–33.57, p<0.001)	18.00 (3.18–101.81, p = 0.001)	10.75 (2.13–54.19, p = 0.007)
**bronchialobstruction**	No	-	-	-
	Yes	2.31 (1.02–5.24, p = 0.045)	-	-
**dissemination**	No	-	-	-
	Yes	1.74 (0.79–3.84, p = 0.169)	-	-
**limfoadenopatia**	No	-	-	-
	Yes	5.81 (2.63–12.82, p<0.001)	-	-
**lungcapacitydecrease**	No	-	-	-
	Yes	2.49 (1.12–5.55, p = 0.025)	-	-
**lungcavitysize**	No cavities	-	-	-
	LTE to 25mm	1.43 (0.59–3.45, p = 0.429)	1.33 (0.43–4.13, p = 0.627)	1.22 (0.45–3.29, p = 0.680)
	More than 25mm	5.42 (1.88–15.62, p = 0.002)	2.34 (0.27–20.00, p = 0.439)	3.55 (1.05–12.01, p = 0.043)
**nodicalcinatum**	No	-	-	-
	Yes	1.96 (0.67–5.71, p = 0.219)	-	-
**plevritis**	No	-	-	-
	Yes	3.35 (1.34–8.38, p = 0.010)	-	-
**pneumothorax**	No	-	-	-
	Yes	8.74 (2.61–29.23, p<0.001)	-	-
**posttbresiduals**	No	-	-	-
	Yes	0.68 (0.16–2.87, p = 0.597)	-	-
**processprevalence**	Less than 2 segments	-	-	-
	2 or more segments	4.17 (1.43–12.16, p = 0.009)	0.51 (0.10–2.52, p = 0.411)	0.71 (0.13–3.79, p = 0.674)
**totalcavernum**	No cavities	-	-	-
	More than 2 cavities	4.62 (1.96–10.88, p<0.001)	-	-
	LTE to 2 cavities	0.79 (0.25–2.48, p = 0.687)	-	-

All radiologist annotations were used for univariate modeling whereas only the top 5 features selected by mutual information with treatment outcome were used for multivariate estimates. For multiple imputation, the MICE algorithm was used with default settings to generate 5 imputed datasets used for calculating estimates that were then pooled for the multiply imputed estimates. Cured and died columns show the number of complete cases having the particular radiologist annotation with percentage of cases with that particular observation having the particular outcome in parenthesis that is available for univariate modeling. HR columns show the hazard ratios for univariate, multivariate, and multiple imputed multivariate estimates respectively with p-values and 95% confidence intervals in parenthesis with upper and lower bounds shown with a dash between. For univariate or multivariate reference levels within a covariate, a dash is used. For variables not used in the multivariate model, a dash is provided. Markers of disease progression such as presence of TB-related annotations in both lungs and cavity size show statistically significant higher hazard ratios for an outcome of died. Glossary: Affectlevel–location of affected lung area; affectpleura—changes in the pleura; bodysite_coding_cd–which lung is the observation located; bronchialobstruction—bronchial obstruction syndrome disorders, dissemination—Diffuse pulmonary nodules detected; limfoadenopatia–greater than 10 mm is considered the upper limit for normal nodes (short transverse diameter); lungcapacitydecrease—reduced lung volumes; lungcavitysize–size of lung cavity; nodalcalcinatum—Nodi Calcinatum detected; plevritis—pleural effusion detected; pneumothorax—Pneumothorax detected; posttbresiduals—Post-TB changes in the lung; processprevalence–prevalence of process in number of segments; totalcavernum–number of cavities; thromboembolismpulmonaryartery—Thromboembolism Of The Pulmonary Artery detected; anomalymediastinumvesselsdevelop—Anomaly Of Mediastinum Vessels Develop detected; shadowpattern–shadowpattern of nodule, node, or infiltrate; affectedsegments–segments of lung that are affected; accumulationcontrast–amount of contrast accumulated.

**Fig 1:**
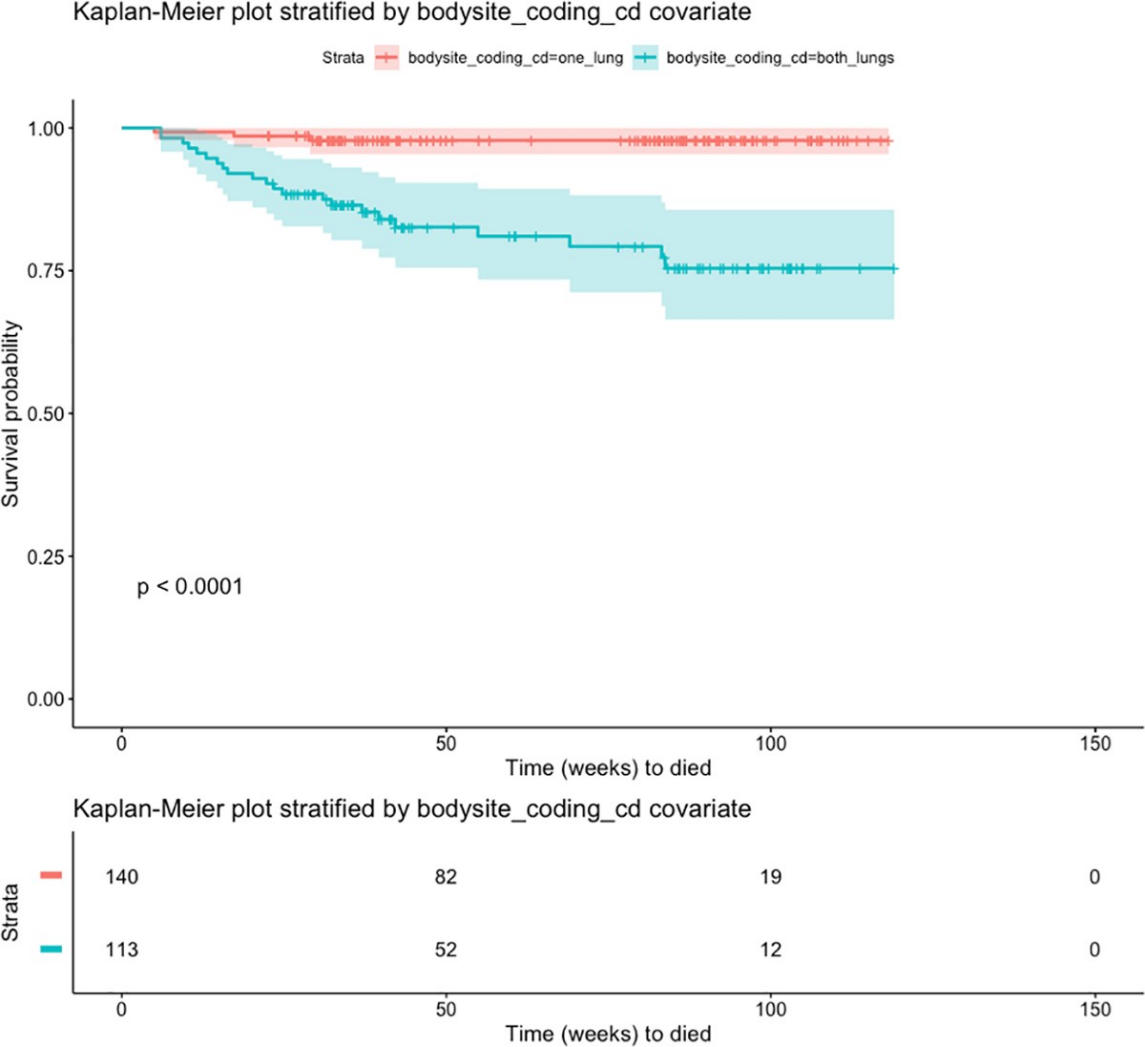
Kaplan-Meier curve showing probability of survival over time stratified by occurrence of radiologist observation in both lungs and only one lung. Shaded area reflects the 95% confidence interval of each survival curve. The table show the number of cases at each time point. P-value shows the log-rank test comparing all survival curves.

**Fig 2:**
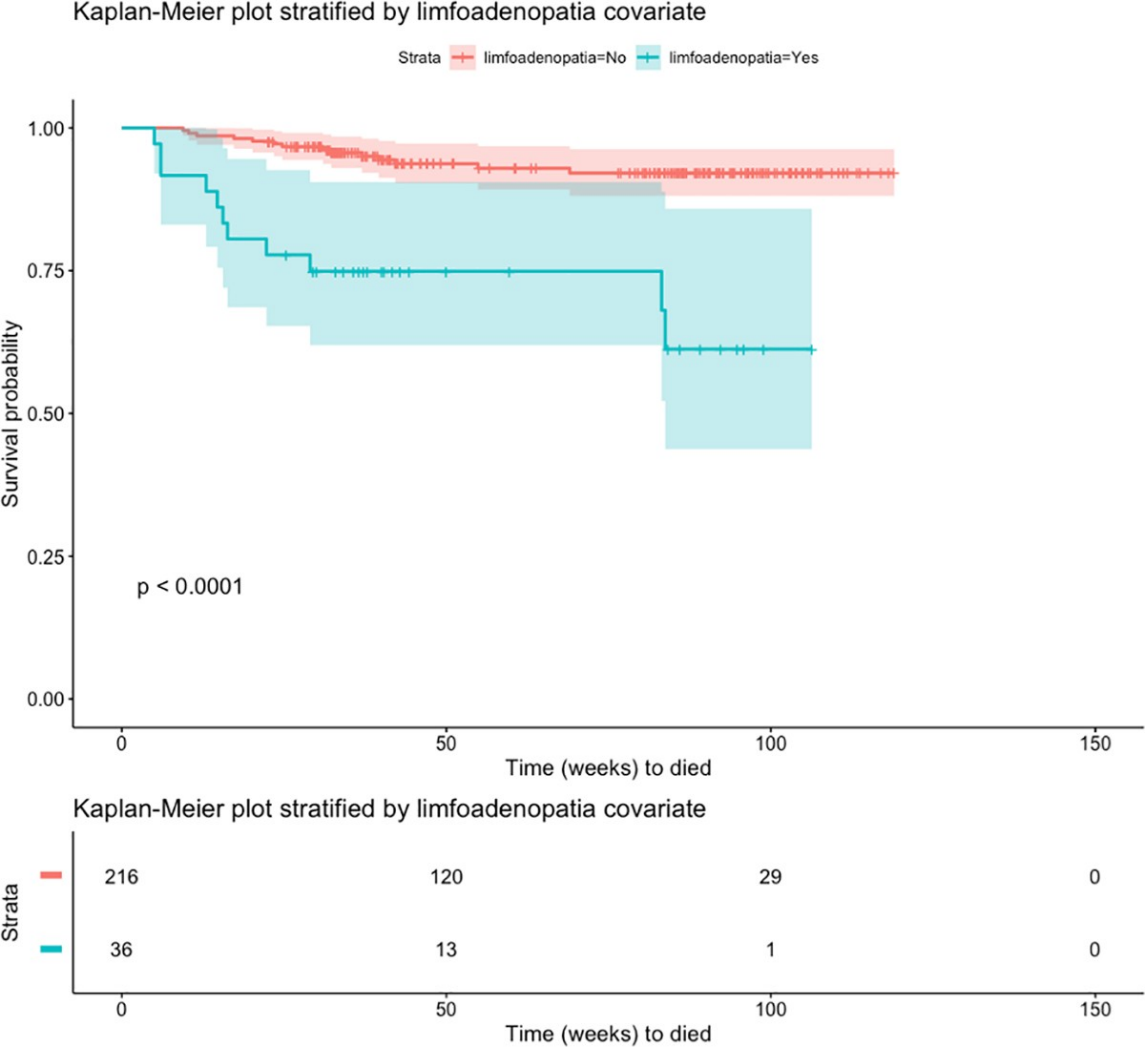
Kaplan-Meier curve showing probability of survival over time stratified by occurrence of radiologist observation of lymphadenopathy. Shaded area reflects the 95% confidence interval of each survival curve. The table show the number of cases at each time point. P-value shows the log-rank test comparing all survival curves.

**Fig 3:**
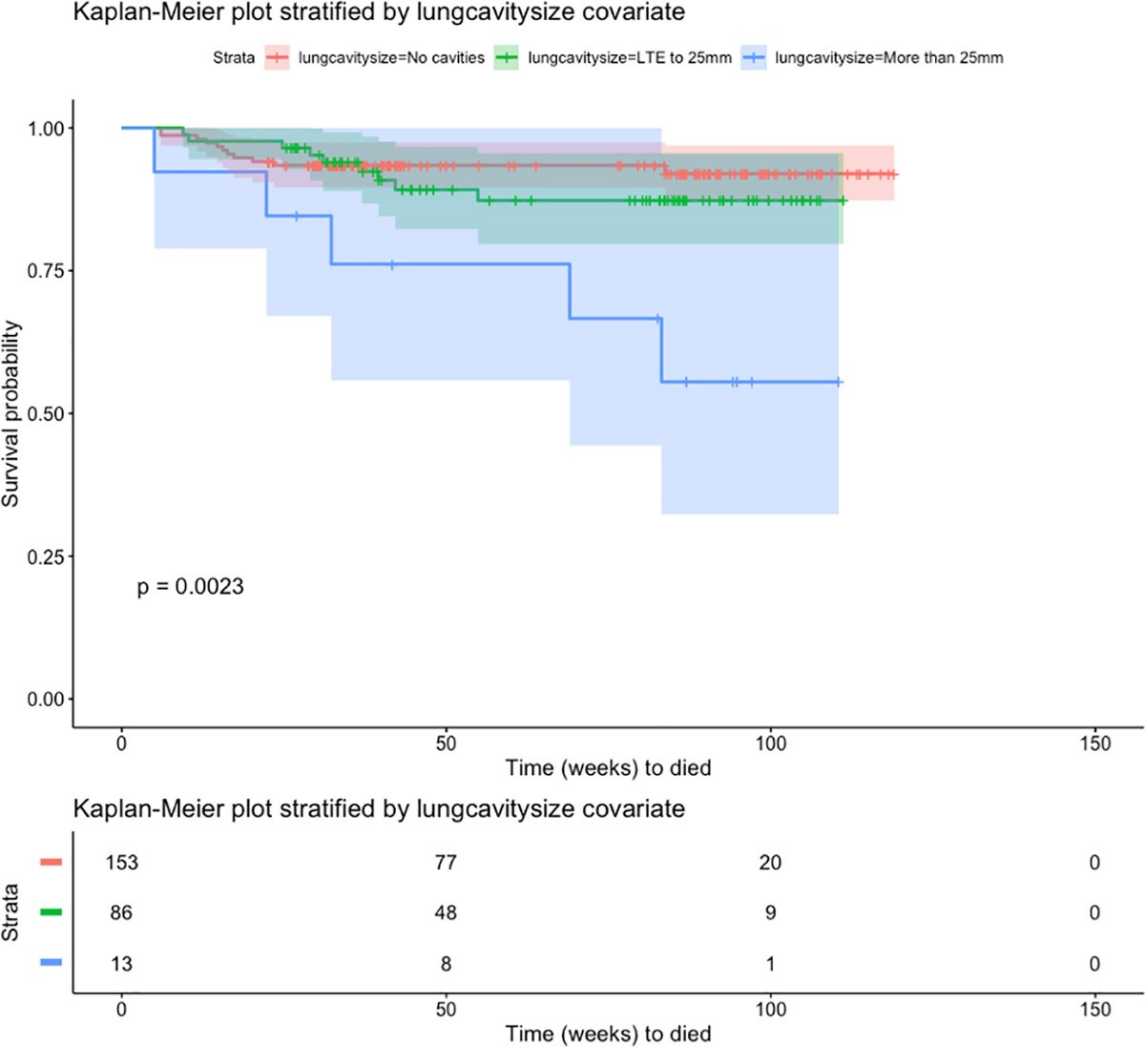
Kaplan-Meier curve showing probability of survival over time stratified by occurrence of radiologist annotation of observation of lung cavity size. Shaded area reflects the 95% confidence interval of each survival curve. The table show the number of cases at each time point. P-value shows the log-rank test comparing all survival curves.

To assess survival probabilities over time, we plotted Kaplan-Meier curves for covariates in which statistically significant differences in odds ratios or hazards ratios were noted such as presence of radiologist annotation in both lungs, presence of swollen lymph nodes, and lung cavity size. Radiologist observations associated with both lungs showed statistically significant differences in the survival curves by log-rank test (Fig 1). The probability of survival for those cases with radiologist observations involving both lungs were lower than those cases with radiologist observations in one lung. Observation of swollen lymph nodes also showed statistically significant differences in the survival curves with cases involving swollen lymph nodes having a decreased probability of survival (Fig 2). Lastly, we observed a larger decrease in survival probability over time in the KM curves where radiologist noted large cavities greater than 25mm in size (Fig 3) although the 95% confidence intervals overlapped suggesting that additional data collection is warranted to increase confidence. Altogether the KM survival curves support the finding from the inferential estimates of the logistic and cox regression models. Biomarkers of disease spread and activity are associated with statistically significant decreased survival probability over time in this cohort.

### Assessing predictive performance of machine learning models

A major goal of the TB Portals program is to improve the underlying data as well as assess analytical approaches that advance knowledge of TB. Mlr3 is an ecosystem of R packages the provide flexible pipelines for a mix and match approach to machine learning similar to the scikit-learn module in python. This philosophy fit our approach as data wrangling steps were done in R and we needed to compare various preprocessing on model performance in an unbiased manner. Furthermore, Mlr3 provides a featureless classifier that only predicts the majority class and reflects a model with limited utility as a control. 25 of 253 cases had the outcome of “died” and we sought to predict this rarer, clinically relevant outcome. We hypothesized that class balancing would improve model performance and tested this hypothesis by comparing model performance of binary classifiers with and without class balancing by fivefold cross validation (Table 3). While no class balancing had higher overall accuracy and sensitivity (optimization led to prediction similar to a featureless model where only cured outcome is predicted), class balancing improved model performance to detect the clinically relevant outcome of died. One can observe the increased performance after incorporation of class balancing or SMOTE preprocessing steps through the relative stability of AUC metric with concordant increases in the balanced accuracy, matthews correlation coefficients, and specificity.

**Table 3:** Comparison of different class balancing approaches on model performance with 5-fold cross-validation on training data.

Preprocessing	Base learner	acc	auc	bacc	bbrier	mcc	sensitivity	specificity
No class balancing	featureless	0.89 +/- 0	0.5 +/- 0	0.5 +/- 0	0.11 +/- 0	0 +/- 0	1 +/- 0	0 +/- 0
No class balancing	kknn	0.87 +/- 0	0.67 +/- 0.07	0.49 +/- 0	0.1 +/- 0.01	-0.06 +/- 0	0.97 +/- 0	0 +/- 0
No class balancing	log_reg	0.87 +/- 0.04	0.79 +/- 0.23	0.49 +/- 0.02	0.11 +/- 0.01	-0.06 +/- 0.04	0.97 +/- 0.04	0 +/- 0
No class balancing	multinom	0.84 +/- 0	0.79 +/- 0.25	0.47 +/- 0	0.11 +/- 0.02	-0.08 +/- 0	0.94 +/- 0	0 +/- 0
No class balancing	ranger	0.89 +/- 0	0.79 +/- 0.16	0.5 +/- 0	0.08 +/- 0.03	0 +/- 0	1 +/- 0	0 +/- 0
Class balancing	featureless	0.58 +/- 0.08	0.5 +/- 0	0.43 +/- 0.03	0.25 +/- 0	-0.08 +/- 0.04	0.62 +/- 0.04	0.33 +/- 0.12
Class balancing	kknn	0.87 +/- 0.08	0.7 +/- 0.1	0.62 +/- 0.04	0.12 +/- 0.06	0.22 +/- 0.19	0.94 +/- 0.09	0.25 +/- 0
Class balancing	log_reg	0.7 +/- 0.07	0.8 +/- 0.12	0.77 +/- 0.04	0.2 +/- 0.04	0.34 +/- 0.05	0.68 +/- 0.09	0.75 +/- 0.37
Class balancing	multinom	0.71 +/- 0.16	0.83 +/- 0.17	0.78 +/- 0.2	0.22 +/- 0.05	0.34 +/- 0.25	0.74 +/- 0.13	1 +/- 0
Class balancing	ranger	0.74 +/- 0.12	0.85 +/- 0.11	0.7 +/- 0.23	0.19 +/- 0.06	0.25 +/- 0.3	0.71 +/- 0.09	0.75 +/- 0.37
SMOTE	featureless	0.89 +/- 0	0.5 +/- 0	0.5 +/- 0	0.11 +/- 0	0 +/- 0	1 +/- 0	0 +/- 0
SMOTE	kknn	0.87 +/- 0.04	0.73 +/- 0.1	0.6 +/- 0.04	0.12 +/- 0.02	0.22 +/- 0.14	0.94 +/- 0.04	0.25 +/- 0
SMOTE	log_reg	0.68 +/- 0.12	0.8 +/- 0.19	0.78 +/- 0.17	0.21 +/- 0.04	0.34 +/- 0.22	0.71 +/- 0.13	0.75 +/- 0.37
SMOTE	multinom	0.68 +/- 0.12	0.79 +/- 0.23	0.78 +/- 0.16	0.23 +/- 0.02	0.34 +/- 0.2	0.71 +/- 0.17	1 +/- 0
SMOTE	ranger	0.68 +/- 0.23	0.77 +/- 0.17	0.62 +/- 0.09	0.19 +/- 0.05	0.22 +/- 0.09	0.74 +/- 0.22	0.67 +/- 0.12

Various model performance metrics such as classification accuracy (acc), AUC (auc), balanced accuracy (bacc), Brier score (bbrier), Matthews correlation coefficient (mcc), sensitivity, and specificity are shown with median +/- MAD for the 5 fold cross-validation results. Preprocessing refers to whether the pipeline included a class balancing step, SMOTE, or no class balancing. Base learner refers to the type of machine learning model used in the pipeline including featureless (only predict most abundant class or random class in case of a tie), log reg (logistic regression), multinom (multinomial log-linear learner via neural networks), ranger (random forest), or kknn (weighted k-nearest neighbor). Metrics of performance are calculated at a probability threshold of 0.5 for determining cured versus died outcome.

Since the relative dates of the initial CTs with available radiologist observations and the treatment end dates associated with each treatment outcome were available from the TB portals data, we modeled the time-to-event from the initial CT to the treatment end date and assessed model performance by the Harrell’s C metric. To do this, the number of weeks from the initial CT with radiologist annotation to treatment period end were calculated and a variety of time-to-event algorithms benchmarked using Mlr3 (Table 4). The cox proportional hazards and tree based time-to-event models demonstrated better performance compared to the Kaplan-Meier (KM) model which takes the survival probability over time of at risk cases. The Kaplan-Meir curve can be considered as a control for model performance where predictive models should perform better than the 0.5 Harrell’s C score of the KM model. The benchmarks from both binary classification and time-to-event analysis establish that the CT annotations contain features that can predict treatment outcome better than control models for the training set and suggest that such predictive performance might translate to unobserved data with similar features.

**Table 4:** Comparison of survival model performance by 5 fold cross-validation on the training data.

Base learner	harrellC
**kaplan**	0.5 +/- 0
**coxph**	0.75 +/- 0.2
**rpart**	0.71 +/- 0.24

Performance of the cox proportional hazards, Kaplan-Meier, and random forest survival models by 5 fold cross-validation using Harrell’s C (harrellC) as the model performance metric. The median 5-fold cross-validation results are shown +/- MAD. Base learner refers to the type of machine learning model used in the pipeline including kaplan (Kaplan-Meier), coxph (cox proportional hazards), or rpart (tree based survival model).

To assess whether the observed training performance translates to performance on similar unobserved data, we held-out a set of 25% of the data constituting the validation set. Mlr3 facilitates nested resampling strategies used in the benchmarking, which should provide an accurate estimate of model performance including preprocessing such as class balancing. The validation set was used to test this theory in practice. Binary classification models trained on the entire 75% of the training dataset used for benchmarking were used for prediction on the 25% held-out validation data. Model predictions were assessed using the same metrics as for training benchmarking (Table 5). The validation model metrics fall into the ranges observed in the training indicating that benchmarking identified performance estimates indicative of actual performance on unobserved data. Class balancing provided improvements to the detection and prediction of an outcome of died either through Mlr3’s default class balancing approach or the use of the SMOTE algorithm. For survival models, model performance on the validation set also showed Harrell’s C scores falling within estimate ranges from the benchmarking results on the training data (Table 6). Altogether, the validation and benchmarking results establish that CT annotations from TB portals are predictive of treatment outcomes and set a reference upon which models incorporating these features can be improved upon henceforth. Nonetheless, these findings need to be considered hypothesis-generating rather than suggesting that actionable steps be taken clinically for patients meeting these criteria given that other observed or unobserved factors could be contributing to the findings.

**Table 5:** Comparison of different class balancing approaches on binary classifier model performance on validation data.

Preprocessing	Base learner	acc	auc	bacc	bbrier	mcc	sensitivity	specificity
**No class balancing**	featureless	0.91	0.50	0.50	0.09	0.00	1.00	0.00
**No class balancing**	kknn	0.91	0.71	0.50	0.09	0.00	1.00	0.00
**No class balancing**	log_reg	0.91	0.72	0.50	0.08	0.00	1.00	0.00
**No class balancing**	multinom	0.91	0.76	0.50	0.08	0.00	1.00	0.00
**No class balancing**	ranger	0.91	0.84	0.50	0.08	0.00	1.00	0.00
**Class balancing**	featureless	0.45	0.50	0.40	0.25	-0.12	0.47	0.33
**Class balancing**	kknn	0.78	0.76	0.66	0.14	0.22	0.81	0.50
**Class balancing**	log_reg	0.67	0.80	0.82	0.20	0.38	0.64	1.00
**Class balancing**	multinom	0.66	0.82	0.81	0.18	0.36	0.62	1.00
**Class balancing**	ranger	0.69	0.82	0.83	0.19	0.39	0.66	1.00
**SMOTE**	featureless	0.09	0.50	0.50	0.91	0.00	0.00	1.00
**SMOTE**	kknn	0.92	0.70	0.58	0.11	0.39	1.00	0.17
**SMOTE**	log_reg	0.53	0.73	0.74	0.24	0.28	0.48	1.00
**SMOTE**	multinom	0.53	0.75	0.74	0.23	0.28	0.48	1.00
**SMOTE**	ranger	0.69	0.74	0.68	0.20	0.22	0.69	0.67

Various model performance metrics such as classification accuracy, AUC, balanced accuracy (bacc), Brier score (bbrier), Matthews correlation coefficient (mcc), sensitivity, and specificity are shown after model prediction on the 25% held out validation data set. Preprocessing refers to whether the pipeline included a class balancing step, SMOTE, or no class balancing. Base learner refers to the type of machine learning model used in the pipeline including featureless (only predict most abundant class or random class in case of a tie), log reg (logistic regression), multinom (multinomial log-linear learner via neural networks), ranger (random forest), or kknn (weighted k-nearest neighbor). Metrics of performance are calculated at a probability threshold of 0.5 for determining cured versus died outcome.

**Table 6:** Comparison of survival model performance on validation data.

Base learner	harrellC
**coxph**	0.78
**kaplan**	0.50
**rpart**	0.68

Harrell’s C (harrellC) metric was used to compare cox proportional hazards, Kaplan-Meier, and random forest survival model performance on the validation data set constituting 25% of the data that was held out to assess performance on data that had not been observed before. Base learner refers to the type of machine learning model used in the pipeline including kaplan (Kaplan-Meier), coxph (cox proportional hazards), or rpart (tree based survival model).

## Discussion

CT imaging of the lung provides an important modality for identifying biomarkers of TB severity and progression as pulmonary abnormalities are a common disease manifestation [16]. The TB portals provides CT images of lungs associated with TB cases along with patient-centric, temporal information that helps to put the image in the context of the real-world clinical journey. We leveraged TB portals data to identify CT images with associated radiologist observations to assess the utility of these observations independently of other case attributes towards risk of poor treatment outcome. While CT images and radiologist observations have been used previously to assess patient treatment outcome [8,9] or response [17], the analysis was done in the context of a clinical trial or study that was limited by the available sample size. The use of real-world data sources such as TB portals can facilitate exploration of the predictors of poor treatment outcome in real-world settings and additional questions can be addressed as more cases are added over time. Real-world evidence can inform clinical practice by exploring the potential of lung CT images as clinical end points or markers of disease severity. Here we demonstrate that for new TB cases, the radiologist observations associated with CT images taken within 60 days of the initial case registration into the database contain statistically significant risk factors associated with poor treatment outcome.

We chose to analyze cured versus died outcomes because they represent the boundaries of the available treatment end points deemed beneficial or adverse from a clinical perspective. We reasoned that such edge cases may contain the greatest differences in radiological signatures with which to assess machine learning models. Nonetheless, this approach has a limitation in that it cannot be used to predict intermediary treatment end points such as failure that fall between the two extremes. Moreover, given differences in treatment efficiencies due to the recommended treatment plans for sensitive or various drug resistant TB subtypes, it would have been beneficial to model the TB subtype as well but the numbers of available cases with the relevant treatment outcomes did not support this approach. Therefore, this analysis did not incorporate TB subtype differences within the modeling meaning it is possible certain aspects of the temporal response to treatment may be affecting model estimates (especially in the time-to-event models). We attempt to limit this potential by selecting CTs around the time of registration to decrease the potential of treatment to affect CT observations. We also observe that the proportions of TB cases by subtype is not statistically significant between those with a treatment outcome of died versus those with a treatment outcome of cured suggesting that the impacts would be modest. As more data is collected increasing the number of cases with the outcomes of interest, it would be interesting to include the subtype of TB as a random effect for instance.

While previous analyses have leveraged TB Portals data to predict treatment outcome using machine learning approaches [18,19], they predicted multiple treatment outcomes that may be challenging for machine learning approaches to delineate (e.g. cured versus failure versus died). Moreover, previous approaches leveraged the entire TB portals case record which include information that is not available at clinically relevant time points such as around the time of the initial diagnosis. The number of CT images or X-ray images taken over the course of the case is an example of information which is only known at the end of treatment. Models generated using all case characteristics may identify such variables as important despite these being of limited clinical utility. For example, poor treatment outcome may be associated with a greater number of medical images simply due to the desire of clinicians to monitor disease progression and treatment response especially in the riskiest cases. Models incorporating these variables may miss other salient variables of clinical relevance. Lastly, prior attempts at analyzing TB portals data do not account for the class imbalancing that can arise despite this being a common issue with predicting biological outcomes.

We observed class imbalance in our analysis as ~10% of selected cases had an outcome of died. This imbalance can adversely affect machine learning algorithms as optimization may select a model that defaults to predicting the most represented class in order to maximize the objective function [20]. Many approaches have been developed including development of machine learning algorithms that can handle class imbalances, sampling techniques to increase the representation of the rarer observations, and techniques that put a higher cost on misclassification of the class of interest. We address the impact of class imbalance by leveraging Mlr3 approaches for handling class imbalance that can be wrapped in a machine learning pipeline for an unbiased assessment on model performance. We observe that not accounting for class balancing led to a high classification accuracy albeit with little difference in performance compared to the featureless model that only predicts the majority class. Such a model would be of limited clinical utility in that less represented outcomes would often be missed. Class balancing is one approach to address this and increase the performance of machine learning models for predicting these rarer, clinically relevant outcomes.

We focus on using radiologist observations of chest CT images at a clinically important time point (close to initial registration of the case into the database) independent of other case characteristics to assess the data’s utility. By focusing on initial time around registration for new cases with a treatment outcome of cured and died and accounting for class imbalancing, we show that radiologist observations are predictive of treatment outcome within the cohort. We identify markers of disease progression and severity including involvement of both lungs, swollen lymph nodes, multiple cavities, and large cavities which are associated with active TB and demonstrate higher risk of poor treatment outcome via inferential statistics. Cavitation in particular has been shown to be associated with a higher baseline load of MTB bacteria [21] and poorer treatment response [8,9,17,22]. As TB portals collects real-world data, we cannot rule out confounding issues such as selection of new cases that were caught later in disease progression, observed differences amongst the subgroups (e.g. drug resistance subtype mentioned prior), and other unobserved variables that may explain these risk profiles. For instance, radiologists independently review CT images by country site and there could be differences in how each approaches the annotations. Nevertheless, for this analysis the majority of the observations were from Belarus suggesting such impacts would be minimal. Collecting additional data to control for these differences by including them in our models as additional covariates or using matching techniques to ensure similar cases characteristics are potential approaches to mitigate potential confounding. Our initial results offer a rationale for these additional data collection efforts given the promising signals we detected amongst the identified outcomes.

Lastly, deep learning and artificial intelligence (AI) are being used extensively for medical image processing to label and annotate features for diagnostic and prognostic purposes. For example, AI approaches have recently been reported to exceed the capability of a radiologist for distinguishing TB from non-TB using chest radiographs. Nevertheless, radiologist observations of medical images are considered the “gold-standard” reference upon which to support AI development [7]. The TB portals database contains reference data that can help to advance AI by providing a radiologist evaluated ground-truth for comparison. By analyzing radiologist observations, we identify potential lung biomarkers that could be considered priorities for automated identification by AI since these biomarkers are most associated with treatment outcome in our cohort. AI could then generate automated features upon which machine learning methods can be applied, risk scores developed, or manual annotations compared. We are cautiously optimistic about the potential of these real-world biomarkers given our best knowledge of the case although we acknowledge the potential impact of other measured or unmeasured variables. Collecting more data that can increase our understanding of the case may be able to improve our confidence. For instance, if we collected medical history at registration, we may be able to better characterize a new case removing any patients with a long history of respiratory symptoms, which suggests significant progression of disease or perhaps a repeat case. The TB portals program is a community resource and is open to collaboration and feedback from researchers to improve the data, tools, and services provided.

## Supporting information

S1 Fig Session information R function call. Specific R packages, version and platform used during the analysis, which is included for reproducibility.
(TIF)

S2 Fig Correlation of CT radiologist observations among complete cases (N = 202). Cases from the cohort that are not missing any features of interest were compared for correlations between covariates and the dependent variable (event). Positive correlations are shown in blue and negative correlations in red. The correlations between event and covariates indicate associations that follow clinical manifestation of disease such as involvement of both lungs, cavity size, number of cavities, and presence of swollen lymph nodes. Glossary: Affectlevel–location of affected lung area; affectpleura—changes in the pleura; bodysite_coding_cd–which lung is the observation located; bronchialobstruction—bronchial obstruction syndrome disorders, dissemination—Diffuse pulmonary nodules detected; limfoadenopatia–greater than 10 mm is considered the upper limit for normal nodes (short transverse diameter); lungcapacitydecrease—reduced lung volumes; lungcavitysize–size of lung cavity; nodalcalcinatum—Nodi Calcinatum detected; plevritis—pleural effusion detected; pneumothorax—Pneumothorax detected; posttbresiduals—Post-tuberculosis changes in the lung; processprevalence–prevalence of process in number of segments; totalcavernum–number of cavities; thromboembolismpulmonaryartery—Thromboembolism Of The Pulmonary Artery detected; anomalymediastinumvesselsdevelop—Anomaly Of Mediastinum Vessels Develop detected; shadowpattern–shadowpattern of nodule, node, or infiltrate; affectedsegments–segments of lung that are affected; accumulationcontrast–amount of contrast accumulated.
(TIF)

S1 Table Case characteristics of the cohort (N = 253). Case characteristics were compared by treatment outcome. P-values were calculated for continuous variables (age_of_onset and bmi) using analysis of variance test. P-values for categorical variables (registration_date, gender, country, and type_of_resistance) were calculated using Chi-squared test.
(XLSX)

S2 Table Comparison of radiologist observations prior to preprocessing. Radiologist observations prior to preprocessing for machine learning were compared by treatment outcome. P-values were calculated for continuous variables using analysis of variance test. P-values for categorical variables were calculated using Chi-squared test. The following variables were dropped from further analysis: Anomalymediastinumvesselsdevelop, shadowpattern, affectlevel, thromboembolismpulmonaryartery, anomalylungdevelop, and accumulationcontrast. The following variables were refactored (S3 Table) to recombine levels: Lungcavitysize, affectlevel, totalcavernum. Glossary: Affectlevel–location of affected lung area; affectpleura—changes in the pleura; bodysite_coding_cd–which lung is the observation located; bronchialobstruction—bronchial obstruction syndrome disorders, dissemination—Diffuse pulmonary nodules detected; limfoadenopatia–greater than 10 mm is considered the upper limit for normal nodes (short transverse diameter); lungcapacitydecrease—reduced lung volumes; lungcavitysize–size of lung cavity; nodalcalcinatum—Nodi Calcinatum detected; plevritis—pleural effusion detected; pneumothorax—Pneumothorax detected; posttbresiduals—Post-tuberculosis changes in the lung; processprevalence–prevalence of process in number of segments; totalcavernum–number of cavities; thromboembolismpulmonaryartery—Thromboembolism Of The Pulmonary Artery detected; anomalymediastinumvesselsdevelop—Anomaly Of Mediastinum Vessels Develop detected; shadowpattern–shadowpattern of nodule, node, or infiltrate; affectedsegments–segments of lung that are affected; accumulationcontrast–amount of contrast accumulated.
(XLSX)

S3 Table CT radiologist annotations observed in the cohort after preprocessing. Radiologist annotations were compared by treatment outcome. P-values for categorical variables were calculated using Chi-squared test. Glossary: Affectlevel–location of affected lung area; affectpleura—changes in the pleura; bodysite_coding_cd–which lung is the observation located; bronchialobstruction—bronchial obstruction syndrome disorders, dissemination—Diffuse pulmonary nodules detected; limfoadenopatia–greater than 10 mm is considered the upper limit for normal nodes (short transverse diameter); lungcapacitydecrease—reduced lung volumes; lungcavitysize–size of lung cavity; nodalcalcinatum—Nodi Calcinatum detected; plevritis—pleural effusion detected; pneumothorax—Pneumothorax detected; posttbresiduals—Post-tuberculosis changes in the lung; processprevalence–prevalence of process in number of segments; totalcavernum–number of cavities; thromboembolismpulmonaryartery—Thromboembolism Of The Pulmonary Artery detected; anomalymediastinumvesselsdevelop—Anomaly Of Mediastinum Vessels Develop detected; shadowpattern–shadowpattern of nodule, node, or infiltrate; affectedsegments–segments of lung that are affected; accumulationcontrast–amount of contrast accumulated.
(XLSX)

S4 Table Proportional hazards test on the multivariate cox proportional hazards model. P-value corresponds to the statistical test of the cox.zph function that demonstrates that no individual variable nor global violates the proportional hazards test.
(XLSX)

S5 Table Patient and condition ids for the cohort used for this analysis. A table of patient and condition ids is provided for the de-identified records that were used for this analysis.
(XLSX)
