# Supplementary figures and images for: Radiologist observations of computed tomography (CT) images predict treatment outcome in TB Portals, a real-world database of tuberculosis (TB) cases
Source: PLoS One. 2021 Mar 17;16(3):e0247906. doi: 10.1371/journal.pone.0247906 (PMC7968673; doi:10.1371/journal.pone.0247906)

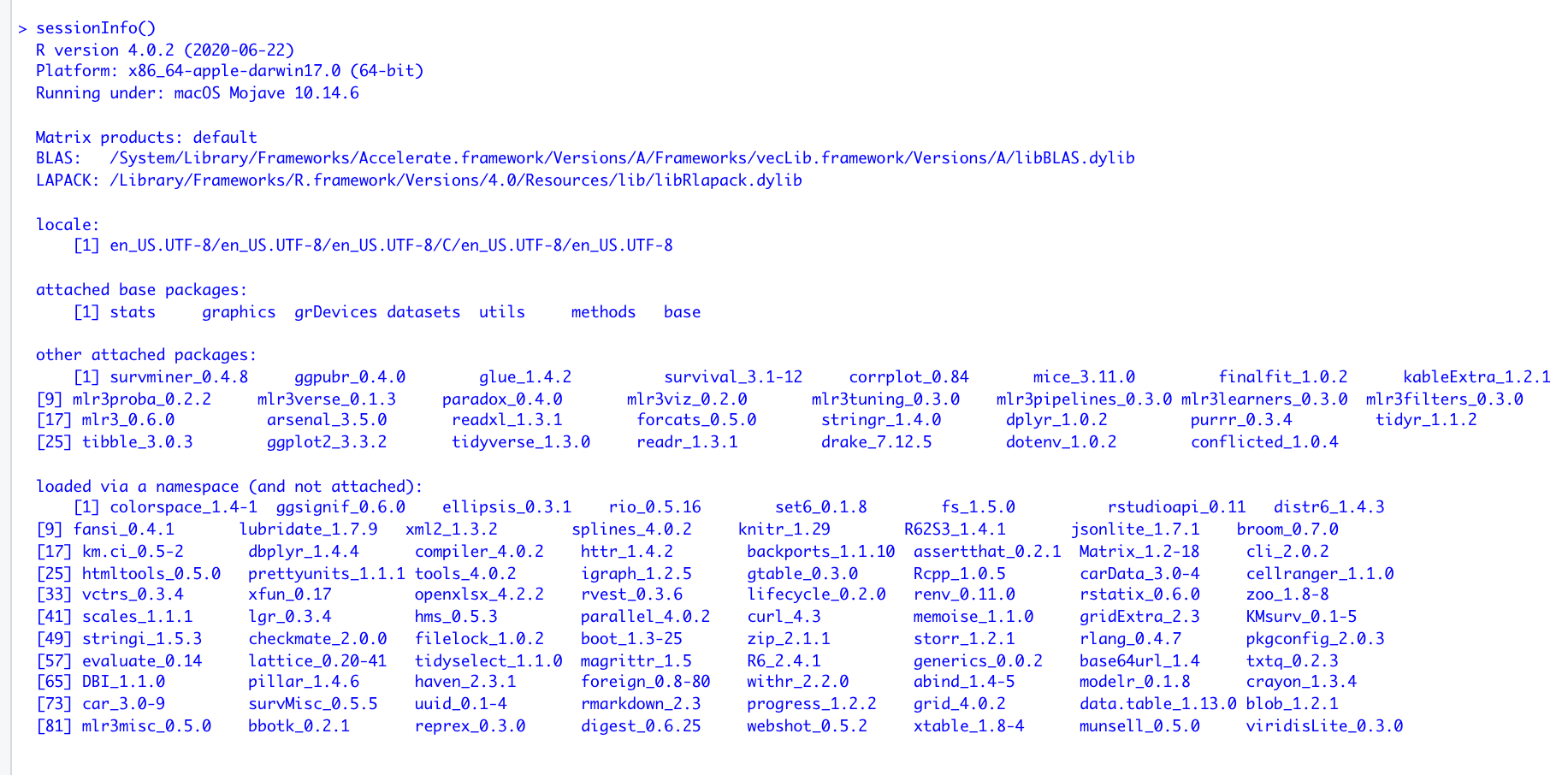

Supplement: S1 Fig — Specific R packages, version and platform used during the analysis, which is included for reproducibility. (TIF) [file pone.0247906.s001.tif]

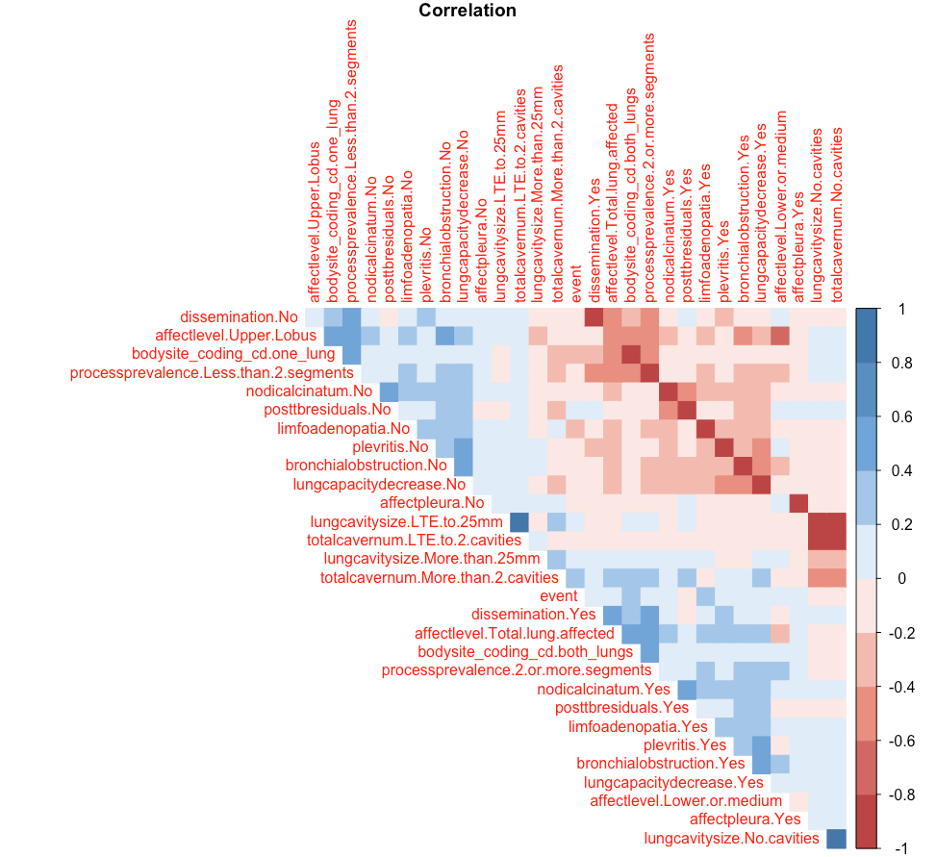

Supplement: S2 Fig — Cases from the cohort that are not missing any features of interest were compared for correlations between covariates and the dependent variable (event). Positive correlations are shown in blue and negative correlations in red. The correlations between event and covariates indicate associations that follow clinical manifestation of disease such as involvement of both lungs, cavity size, number of cavities, and presence of swollen lymph nodes. Glossary: Affectlevel–location of affected lung area; affectpleura—changes in the pleura; bodysite_coding_cd–which lung is the observation located; bronchialobstruction—bronchial obstruction syndrome disorders, dissemination—Diffuse pulmonary nodules detected; limfoadenopatia–greater than 10 mm is considered the upper limit for normal nodes (short transverse diameter); lungcapacitydecrease—reduced lung volumes; lungcavitysize–size of lung cavity; nodalcalcinatum—Nodi Calcinatum detected; plevritis—pleural effusion detected; pneumothorax—Pneumothorax detected; posttbresiduals—Post-tuberculosis changes in the lung; processprevalence–prevalence of process in number of segments; totalcavernum–number of cavities; thromboembolismpulmonaryartery—Thromboembolism Of The Pulmonary Artery detected; anomalymediastinumvesselsdevelop—Anomaly Of Mediastinum Vessels Develop detected; shadowpattern–shadowpattern of nodule, node, or infiltrate; affectedsegments–segments of lung that are affected; accumulationcontrast–amount of contrast accumulated. (TIF) [file pone.0247906.s002.tif]
